# Supplementary material for: The role of the C2A domain of synaptotagmin 1 in asynchronous neurotransmitter release
Source: PLoS One. 2020 May 14;15(5):e0232991. doi: 10.1371/journal.pone.0232991 (PMC7224543; doi:10.1371/journal.pone.0232991)

Raw Blot Images Fig 1

pageruler prestained ladder

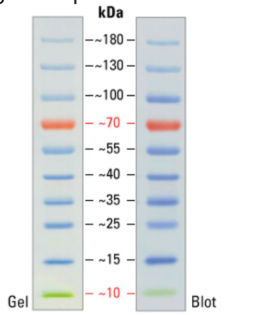

All original blots are included below. Blots were imaged with an Epichemi3 Darkroom with Labworks software. Asterisks denote lanes that were excluded from analysis due to either an inability to quantify or actin control staining that was more than 3 standard deviations above or below the mean actin levels for the blot. L-ladder; 1 - WT, 2 - DN, 3 - DE. Lanes used for the representative blots are outlined in red.

L 1 X 3 1 2 3 X 2 3

70kDa

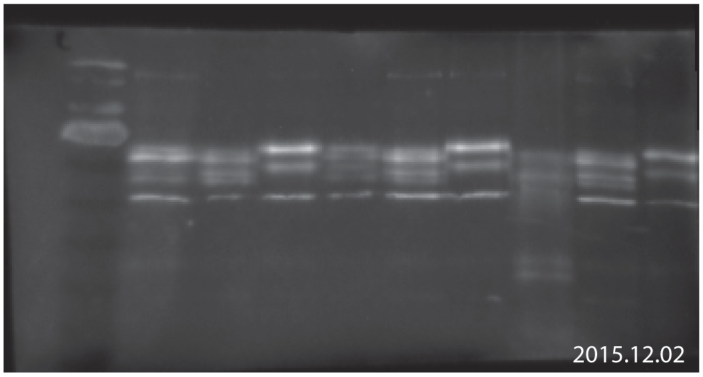

X X 1 2 X 1 2 3 1 L

70kDa

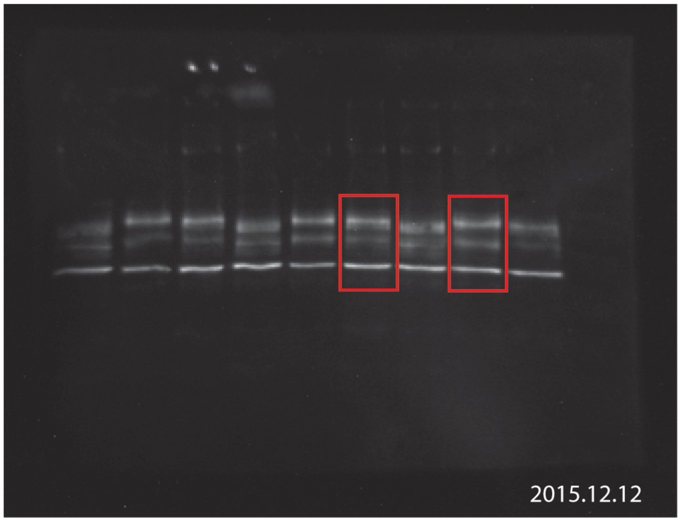

L 1 2 X 1 2 3 1 X 1

70kDa

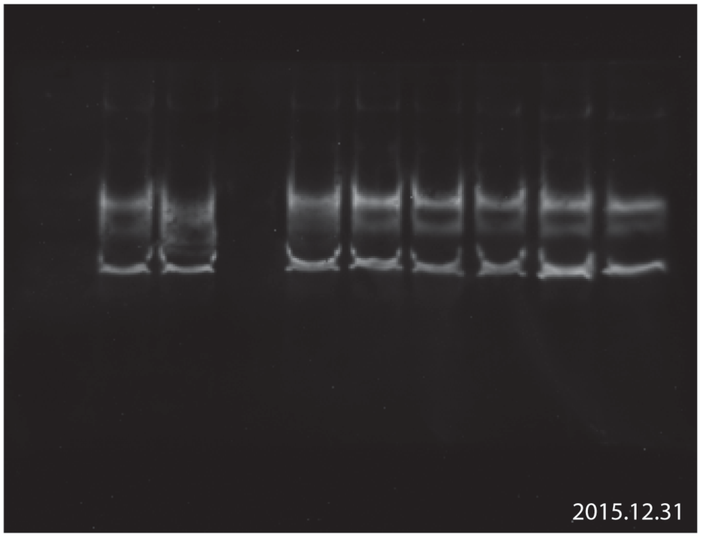

L X 3 X 3 1 3 1 3 3

70kDa

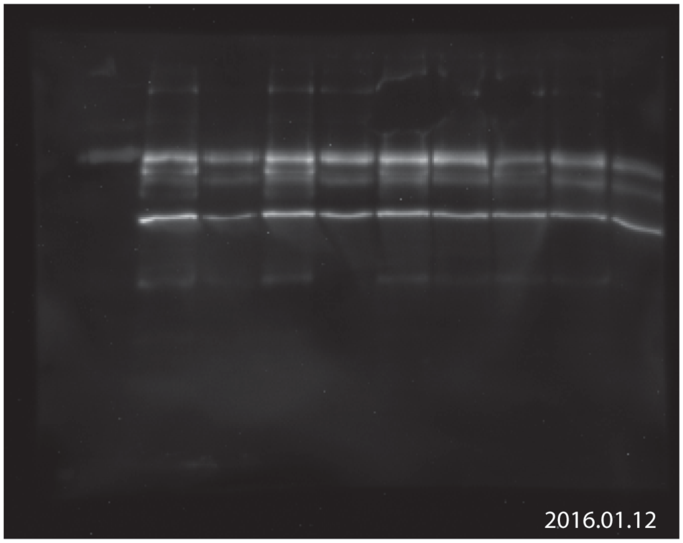

L X 1 X X X 1 X 2 1

70kDa

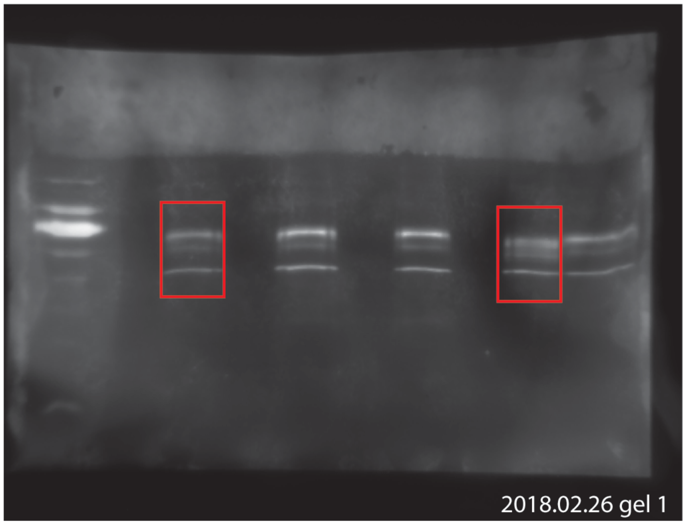

L 1 X 2 X 1 X 2 X 1

70kDa

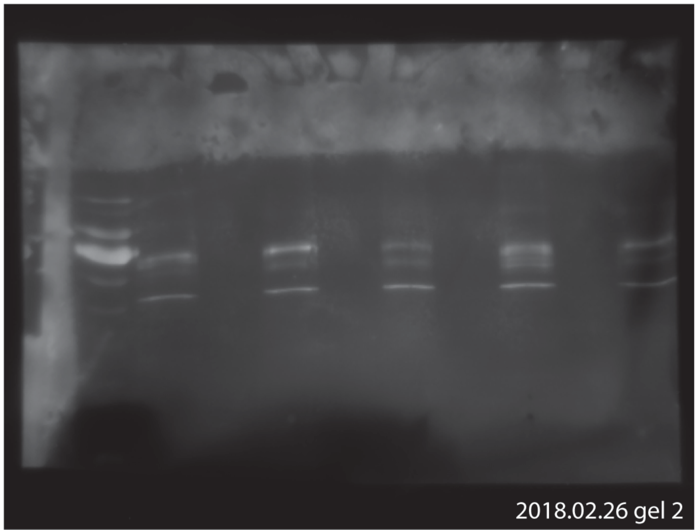

Supplement: S1 Raw Image — (PDF) [file pone.0232991.s001.pdf]
